# The ERK_1/2_ Inhibitor U0126 Attenuates Diabetes-Induced Upregulation of MMP-9 and Biomarkers of Inflammation in the Retina

**DOI:** 10.1155/2013/658548

**Published:** 2013-04-10

**Authors:** Ghulam Mohammad, Mohammad Mairaj Siddiquei, Mohammad Imtiaz Nawaz, Ahmed M. Abu El-Asrar

**Affiliations:** Department of Ophthalmology, College of Medicine, King Saud University, P.O. Box 245, Riyadh 11411, Saudi Arabia

## Abstract

This study was conducted to determine the expression of matrix metalloproteinase-9 (MMP-9) and tissue inhibitor of metalloproteinase-1 (TIMP-1) in a time-dependent manner and the effect of extracellular-signal-regulated kinases-1/2 (ERK_1/2_) inhibition on the expressions of MMP-9, TIMP-1, and inflammatory biomarkers in the retinas of diabetic rats. The expression of MMP-9 was quantified by zymography, and the mRNA level of MMP-9 and TIMP-1 was quantified by RT-PCR. The expression of inducible nitric oxide synthase (iNOS), interleukin-6 (IL-6), and tumor necrosis factor-alpha (TNF-**α**) was examined by Western blot analysis. MMP-9 expression was significantly higher in diabetic rat retinas compared to controls at all time points.TIMP-1 expression was nonsignificantly upregulated at 1week of diabetes and was significantly downregulated at 4 and 12 weeks of diabetes. Intravitreal administration of the ERK_1/2_ inhibitor U0126 prior to induction of diabetes decreased ERK_1/2_ activation, attenuated diabetes-induced upregulation of MMP-9, iNOS, IL-6, and TNF-**α** and upregulated TIMP-1 expression. In MMP-9 knockout mice, diabetes had no effect on retinal iNOS expression and its level remained unchanged. These data provide evidence that ERK_1/2_ signaling pathway is involved in MMP-9, iNOS, IL-6, and TNF-**α** induction in diabetic retinas and suggest that ERK_1/2_ can be a novel therapeutic target in diabetic retinopathy.

## 1. Introduction

Diabetic retinopathy (DR) is the most common microvascular complication of diabetes and remains one of the leading causes of blindness worldwide. DR is characterized by gradual progressive alterations in the retinal microvasculature, leading to loss of retinal capillary cells, disruption of vascular barrier, retinal nonperfusion, and preretinal neovascularization [1–4]. However, the exact molecular mechanisms, which mediate such response, remain largely unknown. In recent years, it has become evident that inflammatory mechanisms play an important role in the pathogenesis of DR, and proinflammatory mediators contribute significantly to the development and progression of DR [5–13]. Inflammation is a multistep process where proteases, growth factors, cytokines, and chemokines are released from retinal cells and interact with each other to promote inflammation in the diabetic retinal microenviroment. In the retina, it was shown that diabetes activates induction of proinflammatory mediators such as monocyte chemoattractant protein-1 (MCP-1) [8], interleukin-6 (IL-6) [9], intercellular adhesion molecule-1 (ICAM-1) [10], inducible nitric oxide synthase (iNOS) [11], tumor necrosis factor-alpha (TNF-*α*) [12], and matrix metalloproteinase-9 (MMP-9) [13]. Recently, much research has focused on MMP-9 because it acts as a potent proinflammatory, proangiogenic and pro-apoptotic factor, and in diabetes, latent MMP-9 is activated in the retina and facilitates retinal capillary cell apoptosis, which is a pathological hallmark of DR development [13–17]. 

Matrix metalloproteinases (MMPs) are a large family of proteinases that remodel extracellular matrix components and play an important role in the regulation of numerous physiological processes including vascular remodeling and angiogenesis. Altered MMPs activities have been implicated in many diseases, including diabetes. Diabetes is believed to stimulate the secretion of several MMPs which participate in both macrovascular and microvascular diseases such as coronary artery disease, peripheral arterial disease, stroke, nephropathy, neuropathy, and retinopathy [18–20]. MMP-9, the largest and most complex member of the MMP family, regulates a variety of cellular functions, including proliferation, differentiation, and angiogenesis. Endogenous tissue inhibitors of MMPs (TIMPs) regulate their activation, and TIMP-1 shows greater preference for MMP-9 than any other MMP [21]. The published literature and our previously published data demonstrated elevated levels of MMP-9 in the epiretinal membranes and vitreous fluid from patients with proliferative diabetic retinopathy (PDR) and increased MMP-9 expression in the retinas of diabetic rodents [13, 15, 16, 22–24]. In addition, our previous data also suggested a pro-apoptotic role of MMP-9 in the pathogenesis of DR [13, 15]. Recently, several studies documented that MMP-9 expression is regulated by extracellular-signal-regulated kinases- (ERK-) dependent pathways [24–27] and in the retina RAF protooncogene serine/threonine-protein kinase- (Raf-) mitogen-activated protein kinase kinase (MEK)-ERK cascade is activated by diabetes [13, 24]. Therefore, inhibitors of Raf-MEK-ERK pathway represent a unique opportunity to prevent MMP-9 induction in the retina induced by diabetes. Previously, it was shown that 1 week after diabetes was induced, the retinal ERK_1/2_, vascular endothelial growth factor (VEGF), ICAM-1, leukostasis, and retinal vascular permeability are significantly upregulated [28–30]. Based on these observations, this study was designed to investigate the potential therapeutic role of ERK_1/2_-specific inhibitor U0126 on the retinas at one week of diabetes in rats. We, therefore, investigated the time course change in the expression of MMP-9 and TIMP-1 in the retinas of diabetic rats and examined the effect of intravitreal administration of U0126 on the expression of MMP-9, TIMP-1, iNOS, IL-6, and TNF-*α* in the diabetic retinas.

## 2. Methods

### 2.1. Animals

*Rats:* Diabetes was induced in rats (male Sprague Dawley, 200–220 g) with streptozotocin (55 mg/kg body weight; Sigma Aldrich, MO, USA). Rats were considered diabetic if their blood glucose was greater than 250 mg/dL. Age-matched normal rats served as control. Blood glucose and weight of animals were measured once a week during the study period. At 1, 4, 8, and 12 weeks after the onset of diabetes, the rats were sacrificed by pentobarbital overdose and the retinas were removed, snap frozen in liquid nitrogen for biochemical measurements. Each group had eight or more rats. The same methods were used for the normal control group. All experiments were performed in accordance to the Association of Research in Vision and Ophthalmology on treatment of Animals in Research and the King Saud University's Animal Care and Use Committee Guidelines.

### 2.2. Intravitreal Injection of the ERK Inhibitor U0126

Sprague Dawley rats (210–225 g) were kept under deep anesthesia, and sterilized solution of U0126 (0.1 mM in phosphate buffer saline (PBS) with a concentration of 5% of dimethyl sulfoxide (DMSO)/5 *μ*L; Santa Cruz Biotechnology, CA, USA) was injected into the vitreous of the right eye. The left eye received 5 *μ*L of sterile solution without U0126 (DMSO to PBS −1 : 19) as previously described [28]. After 24 hours, diabetes was induced with STZ as mentioned above. The animals were sacrificed one week after diabetes was induced, and the retinas were carefully dissected, snap frozen in liquid nitrogen, and stored at −80°C to be analyzed by PCR and western blotting.

### 2.3. Mice

Wild-type (WT) and MMP-9 knockout (MMP-9 KO) mice [31] (a generous gift from Professor Ghislain Opdenakker, Rega Institute for Medical Research, university of Leuven, Belgium) were backcrossed 13 times into C57BL6 background to maximally reduce confounding small but additive genetic influences. To illustrate the latter, only in the 13th backcross generation, the brown fur coat of the knockout mice became black. The WT and KO mice were made diabetic by intraperitoneal injection of streptozotocin for five consecutive days. Mice with blood glucose above 200 mg/dL, 3 days after the last injection of streptozotocin, were considered as diabetic. Age-matched normal WT mice served as controls. To investigate long-term effects on the retina, 3 months after induction of diabetes, mice were sacrificed by pentobarbital overdose, and the retinas were removed from one eye, snap frozen in liquid nitrogen, and stored at −80°C for RNA isolation. All experiments were performed in accordance to the Association of Research in Vision and Ophthalmology (ARVO) and King Saud University's Animal Care and Use Committee Guidelines on handling and treatment of animals for basic science research.

### 2.4. Zymography

Gelatinolytic levels of MMP-9 were estimated in the retina by zymography technique. The samples (50–100 *μ*g protein) were electrophoresed under nonreducing conditions onto 10% SDS-PAGE gels polymerized with 1 mg/mL gelatin. After washing the gel with 2.5% Triton X-100, it was incubated overnight at 37°C in substrate buffer containing 50 mM Tris-HCl, pH 8.0, 5 mM CaCl_2_, and 0.02% NaN_3_. The gel was stained with Coomassie blue stain (0.5% Coomassie blue R-250, 5% methanol, and 10% acetic acid), and this was followed by destaining (5% methanol, 10% acetic acid). The image was taken with the GeneSys (version 1.2.0.0) software on a G:BOX (Syngene, Cambridge, UK) and signal intensity of bands (~92 kDa) quantified using the Gene Tools software (Syngene).

### 2.5. Real-Time Reverse Transcription Polymerase Chain Reaction (RT-PCR)

Total RNA was extracted from retina using TRI reagent (Ambion, TX, USA), according to manufacturer's protocol. cDNA were synthesized from 1 *μ*g RNA, using an high capacity cDNA reverse transcription kit (Applied Biosystem, CA, USA) following manufacturer's instruction. Real-time RT-PCR was performed using a SYBR green PCR master mix. The PCR primers for rats were MMP-9 forward 5′-GCAACGGAGACGGCAAACC-3′ reverse 5′-GACGAAGGGGAAGACGCA-3′; TIMP1 forward 5′-CTGGCATCCTCTTGTTGCT-3′ reverse 5′-CACAGCCAGCACTATAGGTCTTT-3′ and *β*-actin forward 5′-CCTCTATGCCAACACAGTGC-3′ reverse 5′-CATCGTACTCCTGCTTGCTG-3′. The PCR primers for mice were iNOS forward 5′-CACCTTGGAGTTCACCCAGT-3′ reverse 5′-ACCACTCGTACTTGGGATGC-3′ and *β*-actin forward 5′-CCTCTATGCCAACACAGTGC-3′ reverse 5′-CAT CGT ACT CCT GCT TGC TG-3′. The standard PCR conditions included 2 minutes at 50°C and 10 min at 95°C followed by 40 cycles of extension at 95°C for 15 seconds and one minute at 60°C. Threshold lines were automatically adjusted to intersect amplification lines in the linear portion of the amplification curves, and cycles to threshold (Ct) were recorded automatically. Data were normalized with *β*-actin mRNA level (housekeeping gene), and the fold change in gene expression relative to normal was calculated using the ddCt method as previously described [19].

### 2.6. Western Blot Analysis

Retinas were homogenized in a western lysis buffer (30 mM Tris-HCL pH 7.5, 5 mM EDTA, 1% Triton X-100, 250 mM sucrose, 1 mM Sodium vanadate, and protease inhibitor cocktail). The lysate was centrifuged at 14,000 ×g for 15 min at 4°C and the supernatants were collected, and equal amounts of protein (25–40 *μ*g) were subjected to SDS-PAGE and transferred to nitrocellulose membrane. Immunodetection was performed using antibodies against p-ERK_1/2_, iNOS (1 : 1000; Abcam, MA), TNF-*α* (1 : 500; Santa Cruz Biotechnology), and IL-6 (1 : 500; R&D Systems, MN). Membranes were stripped and reprobed with *β*-actin to evaluate the lane-loading control. Bands were visualized using high-performance chemiluminescence machine (G: Box Chemi-XX8 from Syngene, Synoptic Ltd. Cambridge, UK), and the intensities were quantified by using GeneTools software (Syngene by Synoptic Ltd.). 

### 2.7. Statistical Analysis

Each measurement was made in duplicate, and the assays were repeated three or more times. Data are expressed as mean±SD. The Mann-Whitney *U* test was used to compare means from two independent groups. A *P* value less than ≤0.05 indicated statistical significance. SPSS version 12.0 was used for the statistical analyses.

## 3. Results

### 3.1. Time-Dependent Changes in MMP-9 Expression in Diabetic Retinas

**Figure 1:**
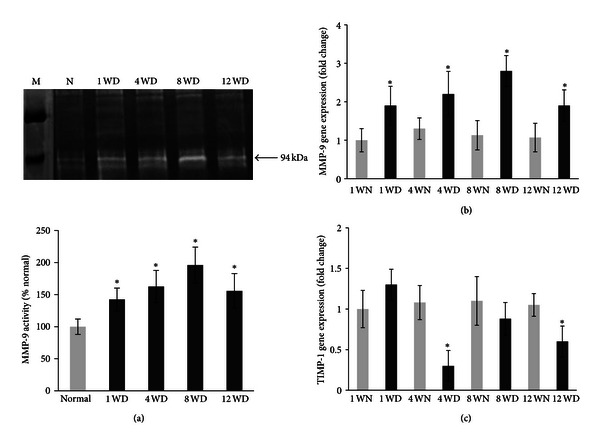
Effect of 1, 4, 8, and 12 weeks of diabetes on retinal MMP-9 and TIMP-1 expression. (a) The gelatinase level of MMP-9 was determined in the retinal homogenate by zymography technique. (b) Gene expressions of MMP-9 and (c) TMP-1 were quantified by RT-PCR using primers given in the Materials and Methods and were adjusted to the mRNA levels of *β*-actin in each sample. Each measurement was performed at least three times. Results are expressed as mean ± s.d. of at least six rats in each group. *P < 0.05 compared with normal rats. M = molecular weight marker; 1 WN, 4 WN, 8 WN, and 12 WN = 1 week normal, 4 weeks normal, 8 weeks normal, and 12 weeks normal rat; 1 WD, 4 WD, 8 WD, and 12 WD = 1 week diabetic, 4 weeks diabetic, 8 weeks diabetic, and 12 weeks diabetic rat.

The MMP-9 gelatinase levels in the retinas of diabetic rats were increased by about 40%, 55%, 85%, and 50% at 1, 4, 8, and 12 weeks, respectively, after the onset of diabetes compared to nondiabetic rats (Figure 1(a)). The relative mRNA levels of MMP-9 were detected by real-time RT-PCR analysis at 1, 4, 8, and 12 weeks after the onset of diabetes. The MMP-9 mRNA levels of the control groups at all time points remained at very constant levels (1 ± 0.3-fold). However, a significant increase (*P* < 0.05) in MMP-9 mRNA levels by about 0.5- to 2-fold was detected in the retinas from 1, 4, 8, and 12 week diabetic rats compared with nondiabetic rats (Figure 1(b)). 

### 3.2. Time-Dependent Changes in TIMP-1 Expression in Diabetic Retinas

As shown in Figure 1(c), the TIMP-1 mRNA levels of the diabetic group was significantly decreased by about 70% and 46% (*P* < 0.01) in the retinas from 4- and 12- week diabetic rats compared to the control group, but there was no significant difference between the control and diabetic animals at week 8. Interestingly, there was a nonsignificant increase in TIMP-1 mRNA in the retinas from 1-week diabetic rats compared to the nondiabetic rats.

### 3.3. U0126 Attenuates Diabetes-Induced ERK_1/2_ Phosphorylation in Diabetic Retinas

**Figure 2:**
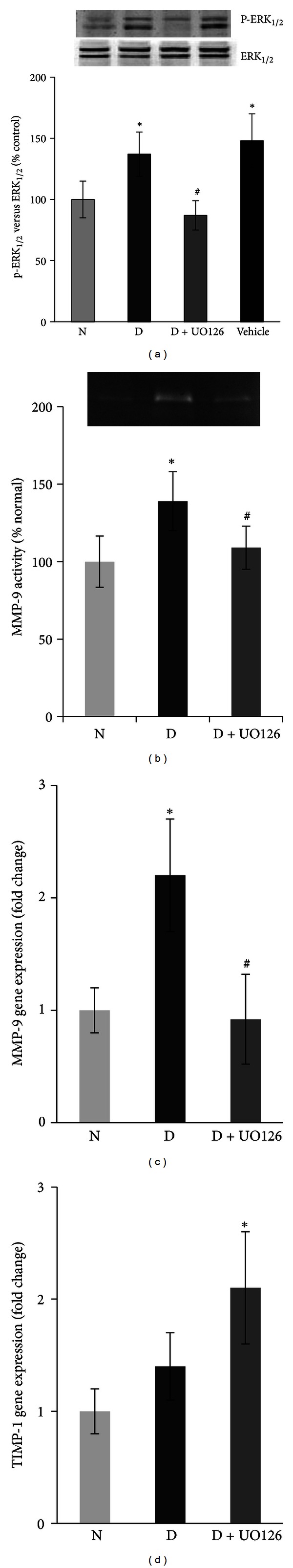
Effect of ERK_1/2_ inhibitor (U0126) on diabetes induced retinal ERK_1/2_ activation and MMP-9 and TIMP-1 expressions. Protein expressions of ERK_1/2_ activation (phosphorylation) were quantified by western blotting using phosphospecific antibody and were adjusted to the protein levels of unphosphorylated antibody in each sample. The expression of (b) MMP-9 gelatinase activity was quantified by using zymography technique. Gene expressions of (c) MMP-9 and (d) TIMP-1 were quantified by RT-PCR using the specific primers and were adjusted to the mRNA levels of *β*-actin in each sample. Each measurement was performed at least three times. Results are expressed as mean ± s.d. of at least six rats in each group. *P < 0.05 compared with normal rats and ^#^ *P* < 0.05 compared to diabetic rats. N, D, and D + U0126 = normal, 1-week diabetic and U0126 pretreated diabetic rat.

U0126 is a potent ERK_1/2_ antagonist and decreases ERK_1/2_ activity in diabetic retina [28]. We employed U0126 to investigate the anti-inflammatory function in the retinas of diabetic rats. Rats that were pretreated with U0126 followed by induction of diabetes showed significant (*P* < 0.05) attenuation of ERK_1/2_ activation as compared to untreated diabetic rats (Figure 2(a)).

### 3.4. Effect of U0126 on the Expression of MMP-9 and TIMP-1

The expression of MMP-9 was significantly attenuated in the U0126-treated diabetic rat retinas as compared to untreated diabetic rats (Figures 2(b) and 2(c)). U0126 pretreatment of the diabetic rats significantly upregulated TIMP-1 expression compared to nondiabetic rats (Figure 2(d)). 

### 3.5. Effect of U0126 on the Expression of the Inflammatory Biomarkers, iNOS, IL-6, and TNF-*α*

**Figure 3:**
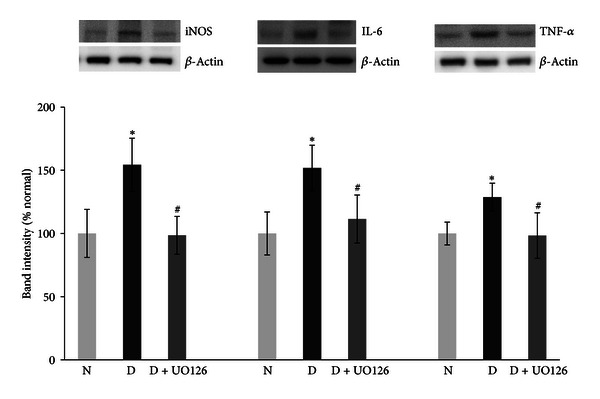
Effect of ERK_1/2_ inhibitor (U0126) on diabetes induced iNOS, IL-6, and TNF-*α* upregulation in diabetic retina. (a) Protein expression of iNOS, (b) IL-6, and (c) TNF-*α* was measured by western blot and *β*-actin was used as housekeeping control. Each measurement was performed at least three times. Results are expressed as mean ± s.d. of at least six rats in each group. *P < 0.05 compared with normal rats and ^#^ *P* < 0.05 compared to diabetic rats. N, D, and D + U0126 = normal, 1 week diabetic and U0126 pretreated diabetic rat.

Diabetes significantly increased the retinal expressions of iNOS by 40%, IL-6 by 60%, and TNF-*α* by 35% as compared to nondiabetic rat retinas. The results as in Figure 3 showed that pretreatment with U0126 significantly attenuated diabetes-induced increase in the expressions of iNOS, IL-6, and TNF-*α* as compared to untreated diabetic rats.

### 3.6. Effect of MMP-9 Inhibition on the mRNA Level of iNOS

**Figure 4:**
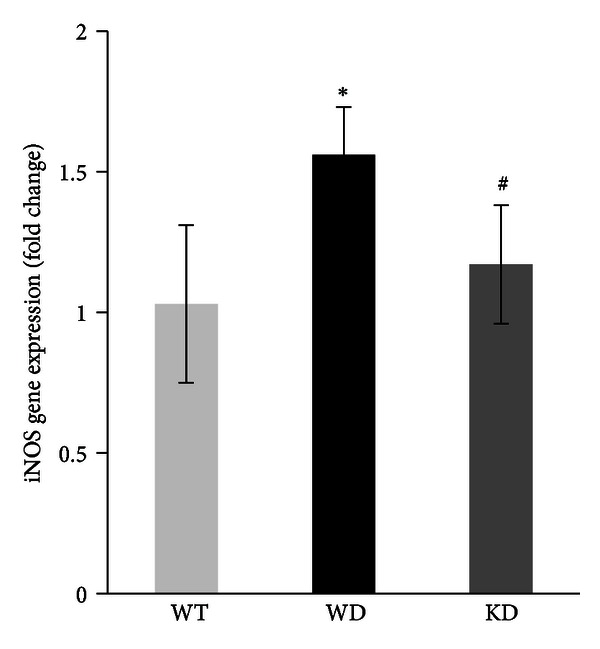
Diabetes does not alter retinal iNOS expression in the mouse lacking MMP-9. Gene expression of iNOS was quantified in the retina of wild-type (WT) mice and MMP-9 KO (KO) mice diabetic for 3 months by real-time quantitative PCR. The mRNA levels of the targeted proteins were adjusted to the levels of *β*-actin in each sample. Results are expressed as mean ± s.d. of at least five to six mice in each group. *P < 0.05 compared with WT nondiabetic mice and ^#^ *P* < 0.05 compared to WT diabetic mice. WT and WD = wild-type nondiabetic and diabetic, respectively, and KD = MMP-9 KO diabetic mice.

Diabetes at ~3 months in WT mice, as expected, increased the expressions of iNOS in the retina by about twofold compared to the WT normal mice. In contrast, in MMP-9 KO mice diabetes had no effect on retinal iNOS expression; the values obtained from diabetic MMP-9 KO mice retina were significantly lower compared to those obtained from WT- diabetic mice (Figure 4).

## 4. Discussion

Inflammation represents a highly coordinated set of events that allow tissues to respond to injury, and it requires the participation of various cell types expressing and reacting to diverse mediators in a sequential manner. In the development of DR, biomarkers of inflammation such as IL-6, iNOS, TNF-*α*, and MMP-9 are elevated in the retina [9, 11–13]. One of the specific objectives of this current study was to determine the role of U0126, an ERK_1/2_ inhibitor, in the regulation of MMP-9 and inflammatory mediators in the diabetic retinas. Salient features of the current study are as follows: (i) in the retina, diabetes upregulates MMP-9 and downregulates TIMP-1 expression in a time-dependent manner; (ii) U0126, a specific inhibitor of ERK_1/2_, attenuates early diabetes-induced upregulation of MMP-9 and enhanced TIMP-1 expression; (iii) U0126 attenuates diabetes-induced upregulation of iNOS, IL6, and TNF-*α* in the retina; (iv) knockdown of MMP-9 prevents diabetes-induced upregulation of iNOS level in the retina. 

 The mitogen-activated protein kinases (MAPKs) play a critical role in the regulation of cell growth and differentiation and in the control of cellular responses to cytokines and stressors. MAPK cascades are a series of cytosolic enzymes that can transmit extracellular signals to the nucleus [32]. These cascades consist of at least three protein kinases that are activated sequentially: a MAPK kinase kinase such as Raf-1 activates a MAPK kinase such as MEK1, which in turn activates a MAPK such as ERK. The activated ERK can translocate to the nucleus [33, 34], where it can phosphorylate or induce transcription factors leading to the activation of genes and the expression of proteins needed for differentiation or proliferation. Growing body of evidence supports the hypothesis that damaging effect of elevated glucose in the retina may, in part, be due to its ability to increase MAPK signaling pathway in the retina [35–38]. ERK_1/2_ is the most extensively characterized member of MAPK family proteins. It plays an important role in cell growth and differentiation, but recent several reports suggested that ERK_1/2_ pathway can also be related to inflammation, apoptosis, and cell injury [39, 40]. A strong relation between ERK_1/2_ activation and MMP-9 is observed in various pathological conditions including diabetes. Our previous work has indicated that H-ras/Raf MEK/ERK_1/2_ mechanisms play a crucial role in the development of diabetic retinopathy [13, 38]. In the present study, we observed that MMP-9 level increases at all time points; however, TIMP-1 expression was downregulated on a time-dependent manner in the retinas of diabetic rats compared to nondiabetic rats. This observation is consistent with previous studies that demonstrated the upregulation of MMP-9 in the retina of diabetic rodents as well as in vitreous of PDR patients [15, 16, 23, 24]. Our findings are also consistent with a previous study that demonstrated decreased TIMP-1 expression in the retina and its microvasculature at both 2 months and 12 months of diabetes [24]. These data suggest a role of TIMP-1-free MMP-9 in the development and progression of DR. Previously, it was demonstrated that the TIMP-1-free MMP-9, readily available for rapid release by neutrophils upon their influx into target tissue, is the major factor determining the high levels of its angiogenic response [41, 42]. We showed here that in the retina, diabetes activates ERK_1/2_ and that UO126 attenuates diabetes-induced activation of ERK_1/2_. Our results are in agreement with a recent report showing increased ERK_1/2_ activity in the retina of one-week diabetic rats compared to normal rats and pretreatment of the retina with U0126 attenuating diabetes-induced ERK_1/2_ activation [28]. Recent studies demonstrated that the activation of ERK_1/2_ plays an essential role in induction of inflammation [40]. ERK_1/2_ activation is required for cytokine signaling, and inhibition of ERK_1/2_ prevents nuclear transcription factor Kappa B (NF-*κ*B) activation [43]. NF-*κ*B has been localized to the inner nuclear layer and ganglion cells of the retina [44], and NF-*κ*B-regulated inflammatory gene products are reported to be upregulated in the retinas during diabetes such as cyclooxygenase-2 [45, 46], iNOS (45,47), ICAM-1 [47, 48], and TNF-*α* [49]. We demonstrated that the therapies that inhibit ERK_1/2_ activation significantly attenuated MMP-9 expression and upregulated TIMP-1 in the retinas of one-week diabetic rats. Similarly, a previous report demonstrated that ERK_1/2_ activation makes a significant contribution to induction of MMP-9 in the rat cortical astrocytes via NF-*κ*B activation [50]. Moreover, selective inhibition of the ERK_1/2_ pathways by U0126 significantly attenuated the recombinant human erythropoietin-induced MMP-9 secretion in mouse brain endothelial cells and neural progenitor cells [51]. iNOS, IL-6, and TNF-*α* are known to be important inflammatory mediators [52]. 

Strong evidence indicates that chronic low-grade inflammation is implicated in the pathogenesis of diabetic retinopathy. Diabetic retinal vascular leakage, capillary nonperfusion, and endothelial cell damage are associated with leukocyte recruitment and adhesion to the retinal vasculature, findings that correlate with the increased expression of ICAM-1 and the leukocyte integrin CD18. Inhibition of ICAM-1 activity in animals deficient in the gene encoding for ICAM-1 or by a neutralizing antibody suppresses both retinal leukostasis and vascular leakage [10, 53]. The causal relationship between inflammation and angiogenesis is now widely accepted [54]. Previously, various studies have documented that diabetes enhanced the production of inflammatory mediators such as iNOS, IL6, and TNF-*α* in the retina [9, 11, 45, 49, 55]. In agreement with these studies, we found a significant upregulation of iNOS, IL6, and TNF-*α* in one-week diabetic rat retinas. In addition, we also demonstrated here that inhibition of ERK_1/2_ by U0126 significantly ameliorates diabetes-induced upregulation of iNOS, IL6, and TNF-*α* in the retina. Recent several reports demonstrated the beneficial effect of ERK_1/2_ inhibition on various inflammatory parameters and on the production of inflammatory cytokines [56, 57]. Maddahi and Edvinsson demonstrated that U0126 significantly inhibits the iNOS, IL-6, and TNF-*α* secretion in rat model of cerebral ischemia [58]. The expression of iNOS in the retina of diabetic mice with manipulated MMP-9 gene remains normal. Similarly, a previous report demonstrated that inhibition of MMP-9 suppresses lipopolysaccharide-induced expression of proinflammatory cytokines and iNOS in microglia [59].

In conclusion, these results indicate that ERK_1/2_ pathway is an upstream signal for MMP-9 production and induction of inflammation in the diabetic retina, and targeting ERK_1/2_ pathway can be a novel therapeutic strategy for the treatment of DR.

## References

[1] Hammes HP, Feng Y, Pfister F, Brownlee M (2011). Diabetic retinopathy: targeting vasoregression. *Diabetes*.

[2] Calcutt NA, Cooper ME, Kern TS, Schmidt AM (2009). Therapies for hyperglycaemia-induced diabetic complications: from animal models to clinical trials. *Nature Reviews Drug Discovery*.

[3] Frank RN (2004). Diabetic retinopathy. *The New England Journal of Medicine*.

[4] Gariano RF, Gardner TW (2005). Retinal angiogenesis in development and disease. *Nature*.

[5] El-Asrar AM (2012). Role of inflammation in the pathogenesis of diabetic retinopathy. *Middle East African Journal of Ophthalmology*.

[6] Rangasamy S, McGuire PG, Das A (2012). Diabetic retinopathy and inflammation: novel therapeutic targets. *Middle East African Journal of Ophthalmology*.

[7] Mohammad G, Siddiquei MM, Othman A, Al-Shabrawey M, Abu El-Asrar AM (2012). . High-mobility group box-1 protein activates inflammatory signaling pathway components and disrupts retinal vascular-barrier in the diabetic retina. *Experimental Eye Research*.

[8] Abu El-Asrar AM, Struyf S, Kangave D, Geboes K, van Damme J (2006). Chemokines in proliferative diabetic retinopathy and proliferative vitreoretinopathy. *The European Cytokine Network*.

[9] Arjamaa O, Pöllönen M, Kinnunen K, Ryhänen T, Kaarniranta K (2011). . Increased IL-6 levels are not related to NF-*κ*B or HIF-1*α* transcription factors activity in the vitreous ofproliferative diabetic retinopathy. *Journal of Diabetes and Its Complications*.

[10] Joussen AM, Poulaki V, Le ML (2004). A central role for inflammation in the pathogenesis of diabetic retinopathy. *FASEB Journal*.

[11] Abu El-Asrar AM, Desmet S, Meersschaert A, Dralands L, Missotten L, Geboes K (2001). Expression of the inducible isoform of nitric oxide synthase in the retinas of human subjects with diabetes mellitus. *American Journal of Ophthalmology*.

[12] Demircan N, Safran BG, Soylu M, Ozcan AA, Sizmaz S (2006). Determination of vitreous interleukin-1 (IL-1) and tumour necrosis factor (TNF) levels in proliferative diabetic retinopathy. *Eye*.

[13] Mohammad G, Kowluru RA (2012). Diabetic retinopathy and signaling mechanism for activation of matrix metalloproteinase-9. *Journal of Cellular Physiology*.

[14] Yar AS, Menevse S, Dogan I (2012). Investigation of ocular neovascularization-related genes and oxidative stress in diabetic rat eye tissues after resveratrol treatment. *Journal of Medicinal Food*.

[15] Kowluru RA, Mohammad G, dos Santos JM, Zhong Q (2011). Abrogation of MMP-9 gene protects against the development of retinopathy in diabetic mice by preventing mitochondrial damage. *Diabetes*.

[16] Descamps FJ, Martens E, Kangave D (2006). The activated form of gelatinase B/matrix metalloproteinase-9 is associated with diabetic vitreous hemorrhage. *Experimental Eye Research*.

[17] Giebel SJ, Menicucci G, McGuire PG, Das A (2005). Matrix metalloproteinases in early diabetic retinopathy and their role in alternation of the blood-retinal barrier. *Laboratory Investigation*.

[18] Nagase H, Woessner JF (1999). Matrix metalloproteinases. *Journal of Biological Chemistry*.

[19] Uemura S, Matsushita H, Li W (2001). Diabetes mellitus enhances vascular matrix metalloproteinase activity role of oxidative stress. *Circulation Research*.

[20] Kadoglou NP, Daskalopoulou SS, Perrea D, Liapis CD (2005). Matrix metalloproteinases and diabetic vascular complications. *Angiology*.

[21] Cruz-Munoz W, Khokha R (2008). The role of tissue inhibitors of metalloproteinases in tumorigenesis and metastasis. *Critical Reviews in Clinical Laboratory Sciences*.

[22] Jin M, Kashiwagi K, Iizuka Y, Tanaka Y, Imai M, Tsukahara S (2001). Matrix metalloproteinases in human diabetic and nondiabetic vitreous. *Retina*.

[23] Abu El-Asrar AM, Dralands L, Veckeneer M (1998). Gelatinase B in proliferative vitreoretinal disorders. *The American Journal of Ophthalmology*.

[24] Kowluru RA (2010). Role of matrix metalloproteinase-9 in the development of diabetic retinopathy and its regulation by H-Ras. *Investigative Ophthalmology and Visual Science*.

[25] Liu P, Wilson MJ (2012). miR-520c and miR-373 upregulate MMP9 expression by targeting mTOR and SIRT1, and activate the Ras/Raf/MEK/Erk signaling pathway and NF-*κ*B factor in human fibrosarcoma cells. *Journal of Cellular Physiology*.

[26] Lakka SS, Jasti SL, Gondi C (2002). Downregulation of MMP-9 in ERK-mutated stable transfectants inhibits glioma invasion *in vitro*. *Oncogene*.

[27] Cho A, Graves J, Reidy MA (2000). Mitogen-activated protein kinases mediate matrix metalloproteinase-9 expression in vascular smooth muscle cells. *Arteriosclerosis, Thrombosis, and Vascular Biology*.

[28] Ye X, Xu G, Chang Q (2010). ERK1/2 signaling pathways involved in VEGF release in diabetic rat retina. *Investigative Ophthalmology and Visual Science*.

[29] Barouch FC, Miyamoto K, Allport JR (2000). Integrin-mediated neutrophil adhesion and retinal leukostasis in diabetes. *Investigative Ophthalmology and Visual Science*.

[30] Qaum T, Xu Q, Joussen AM (2001). VEGF-initiated blood-retinal barrier breakdown in early diabetes. *Investigative Ophthalmology and Visual Science*.

[31] Dubois B, Masure S, Hurtenbach U (1999). Resistance of young gelatinase B-deficient mice to experimental autoimmune encephalomyelitis and necrotizing tail lesions. *Journal of Clinical Investigation*.

[32] Neary JT (2007). MARK cascades in cell growth and death. *News in Physiological Sciences*.

[33] Chen RH, Sarnecki C, Blenis J (1992). Nuclear localization and regulation of erk- and rsk-encoded protein kinases. *Molecular and Cellular Biology*.

[34] Lenormand P, Sardet C, Pages G, L’Allemain G, Brunet A, Pouyssegur J (1993). Growth factors induce nuclear translocation of MAP kinases (p42(mapk) and p44(mapk)) but not their activator MAP kinase kinase (p45(mapkk)) in fibroblasts. *Journal of Cell Biology*.

[35] Abu El-Asrar AM, Dralands L, Missotten L, Al-Jadaan IA, Geboes K (2004). Expression of apoptosis markers in the retinas of human subjects with diabetes. *Investigative Ophthalmology and Visual Science*.

[36] Khan ZA, Chakrabarti S (2007). Cellular signaling and potential new treatment targets in diabetic retinopathy. *Experimental Diabetes Research*.

[37] Mohammad G, Siddiquei MM (2012). Role of matrix metalloproteinase-2 and -9 in the development of diabetic retinopathy. *Journal of Ocular Biology, Diseases, and Informatics*.

[38] Mohammad G, Kowluru RA (2011). The role of Raf-1 kinase in diabetic retinopathy. *Expert Opinion on Therapeutic Targets*.

[39] Jo SK, Cho WY, Sung SA, Kim HK, Won NH (2005). MEK inhibitor, U0126, attenuates cisplatin-induced renal injury by decreasing inflammation and apoptosis. *Kidney International*.

[40] Junttila MR, Li SP, Westermarck J (2008). Phosphatase-mediated crosstalk between MAPK signaling pathways in the regulation of cell survival. *FASEB Journal*.

[41] Ardi VC, Kupriyanova TA, Deryugina EI, Quigley JP (2007). Human neutrophils uniquely release TIMP-free MMP-9 to provide a potent catalytic stimulator of angiogenesis. *Proceedings of the National Academy of Sciences of the United States of America*.

[42] Ardi VC, van den Steen PE, Opdenakker G, Schweighofer B, Deryugina EI, Quigley JP (2009). Neutrophil MMP-9 proenzyme, unencumbered by TIMP-1, undergoes efficient activation in vivo and catalytically induces angiogenesis via a basic fibroblast growth factor (FGF-2)/FGFR-2 pathway. *Journal of Biological Chemistry*.

[43] Kim SD, Yang SI, Kim HC, Shin CY, Ko KH (2007). Inhibition of GSK-3*β* mediates expression of MMP-9 through ERK_1/2_ activation and translocation of NF-*κ*B in rat primary astrocyte. *Brain Research*.

[44] Zheng L, Howell SJ, Hatala DA, Huang K, Kern TS (2007). Salicylate-based anti-inflammatory drugs inhibit the early lesion of diabetic retinopathy. *Diabetes*.

[45] Du Y, Sarthy VP, Kern TS (2004). Interaction between NO and COX pathways in retinal cells exposed to elevated glucose and retina of diabetic rats. *American Journal of Physiology—Regulatory Integrative and Comparative Physiology*.

[46] Carmo A, Cunha-Vaz JG, Carvalho AP, Lopes MC (2000). Effect of cyclosporin-A on the blood-retinal barrier permeability in streptozotocin-induced diabetes. *Mediators of Inflammation*.

[47] Zheng L, Szabó C, Kern TS (2004). Poly(ADP-ribose) polymerase is involved in the development of diabetic retinopathy via regulation of nuclear factor-*κ*B. *Diabetes*.

[48] Joussen AM, Poulaki V, Qin W (2002). Retinal vascular endothelial growth factor induces intercellular adhesion molecule-1 and endothelial nitric oxide synthase expression and initiates early diabetic retinal leukocyte adhesion in vivo. *The American Journal of Pathology*.

[49] Joussen AM, Poulaki V, Mitsiades N (2002). Nonsteroidal anti-inflammatory drugs prevent early diabetic retinopathy via TNF-alpha suppression. *The FASEB Journal*.

[50] Arai K, Lee SR, Lo EH (2003). Essential role for ERK mitogen-activated protein kinase in matrix metalloproteinase-9 regulation in rat cortical astrocytes. *GLIA*.

[51] Wang L, Zheng GZ, Rui LZ (2006). Matrix metalloproteinase 2 (MMP2) and MMP9 secreted by erythropoietin- activated endothelial cells promote neural progenitor cell migration. *Journal of Neuroscience*.

[52] Young HR, Chung CP, Oeser A (2009). Inflammatory mediators and premature coronary atherosclerosis in rheumatoid arthritis. *Arthritis Care and Research*.

[53] Miyamoto K, Khosrof S, Bursell SE (1999). Prevention of leukostasis and vascular leakage in streptozotocin-induced diabetic retinopathy via intercellular adhesion molecule-1 inhibition. *Proceedings of the National Academy of Sciences of the United States of America*.

[54] van Beijnum JR, Buurman WA, Griffioen AW (2008). Convergence and amplification of toll-like receptor (TLR) and receptor for advanced glycation end products (RAGE) signaling pathways via high mobility group B1 (HMGB1). *Angiogenesis*.

[55] Ellis EA, Guberski DL, Hutson B, Grant MB (2002). Time course of NADH oxidase, inducible nitric oxide synthase and peroxynitrite in diabetic retinopathy in the BBZ/Wor rat. *Nitric Oxide*.

[56] Gao B, Calhoun K, Fang D (2006). The proinflammatory cytokines IL-1*β* and TNF-*α* induce the expression of Synoviolin, an E3 ubiquitin ligase, in mouse synovial fibroblasts via the Erk1/2-ETS1 pathway. *Arthritis Research and Therapy*.

[57] Firestein GS, Manning AM (1999). Signal transduction and transcription factors in rheumatic disease. *Arthritis & Rheumatism*.

[58] Maddahi A, Edvinsson L (2010). Cerebral ischemia induces microvascular pro-inflammatory cytokine expression via the MEK/ERK pathway. *Journal of Neuroinflammation*.

[59] Woo MS, Park JS, Choi IY, Kimf WK, Kim HS (2008). Inhibition of MMP-3 or -9 suppresses lipopolysaccharide-induced expression of proinflammatory cytokines and iNOS in microglia. *Journal of Neurochemistry*.

